# Silencing ANGPT2 alleviates ulcerative colitis by regulating autophagy-mediated NLRP3 inflammasome inactivation via the mTOR signaling pathway

**DOI:** 10.1590/1414-431X2024e13379

**Published:** 2024-05-20

**Authors:** Xiaojun Wang, Jian Huang, Jia Liu, Yujie Sun, Xinyi Feng, Yansheng Jin, Weigang Zhou

**Affiliations:** 1 Department of Laboratory Medicine, Suzhou Wuzhong People's Hospital, Suzhou, Jiangsu, China; 2 Suzhou Key Laboratory for Medical Biotechnology, Suzhou Vocational Health College, Suzhou, Jiangsu, China; 3 Department of Gastroenterology, Suzhou Wuzhong People's Hospital, Suzhou, Jiangsu, China

**Keywords:** Ulcerative colitis, ANGPT2, Autophagy, NLRP3 inflammasome, mTOR signaling pathway

## Abstract

Ulcerative colitis (UC) is a difficult intestinal disease characterized by inflammation, and its mechanism is complex and diverse. Angiopoietin-like protein 2 (ANGPT2) plays an important regulatory role in inflammatory diseases. However, the role of ANGPT2 in UC has not been reported so far. After exploring the expression level of ANGPT2 in serum of UC patients, the reaction mechanism of ANGPT2 was investigated in dextran sodium sulfate (DSS)-induced UC mice. After ANGPT2 expression was suppressed, the clinical symptoms and pathological changes of UC mice were detected. Colonic infiltration, oxidative stress, and colonic mucosal barrier in UC mice were evaluated utilizing immunohistochemistry, immunofluorescence, and related kits. Finally, western blot was applied for the estimation of mTOR signaling pathway and NLRP3 inflammasome-related proteins. ANGPT2 silencing improved clinical symptoms and pathological changes, alleviated colonic inflammatory infiltration and oxidative stress, and maintained the colonic mucosal barrier in DSS-induced UC mice. The regulatory effect of ANGPT2 on UC disease might occur by regulating the mTOR signaling pathway and thus affecting autophagy-mediated NLRP3 inflammasome inactivation. ANGPT2 silencing alleviated UC by regulating autophagy-mediated NLRP3 inflammasome inactivation via the mTOR signaling pathway.

## Introduction

As a common and clinically difficult intestinal disease, ulcerative colitis (UC), whose prevalence is on the rise, is predominantly distinguished by persistent and diffuse inflammation with clinical manifestations such as diarrhea, abdominal pain, and other symptoms. (1). UC has a long treatment period and is usually difficult to cure, increasing the risk of colon cancer (2). In view of this, investigating the pathogenesis of UC and finding promising therapeutic targets for UC are of great necessity.

Angiopoietin-like protein 2 (ANGPT2) is a 496-amino-acid long protein with a COOH terminal fibrin-like domain, an NH2 terminal helical coil domain, and a secreted signal peptide (3). ANGPT2 can mediate inflammation as well as atherosclerosis (4). A previous study has shown that ANGPT2 was identified as an important mediator of renal fibrosis and autophagy in diabetic nephropathy, and its knockdown increased autophagy and reduced renal fibrosis by activating the MEK/ERK/Nrf-1 pathway (5). RNA-seq was performed on inflamed and non-inflamed intestinal mucosa in 13 patients with Crohn's disease and on sex-matched normal mucosa of 13 healthy controls, and ANGPT2 expression was found to be higher in inflamed intestinal mucosa than that in non-inflamed intestinal mucosa and normal mucosa (6). ANGPT2 silencing improved LPS-induced intestinal epithelial cell (necrotizing enterocolitis) inflammation and barrier dysfunction via blocking Notch signaling pathway (7). However, the role that ANGPT2 plays in UC is obscure.

Therefore, in this paper, we discussed the regulatory role and mechanism of ANGPT2 in UC, hoping to shed novel insights into the treatment of UC.

## Material and Methods

### Samples from patients

Blood samples were collected from UC patients (n=10) and healthy controls (n=10) from Suzhou Wuzhong People's Hospital. All patients provided written informed consent approved by the medical ethics review boards of Suzhou Wuzhong People's Hospital (number: 2023ky030) prior to inclusion in this study.

### UC mouse model

After one week of adaptation, male C57BL/6 mice (6-8 weeks of age, 20±2 g) were randomly divided into four groups (n=5/group): control group, dextran sodium sulfate (DSS) group, DSS + sh-NC (negative control) group, and DSS + sh-ANGPT2 group. Mice in the DSS group were fed normally and given 3% DSS diluted in water for 7 days (8). Mice in the DSS + sh-ANGPT2 group received 3% DSS and were injected with adeno-associated virus (AAV) shRNA targeting ANGPT2 (sh-ANGPT2) into the tail vein at the same time. Seven days later, the mice were euthanized by cervical vertebra dislocation. Serum, colon tissues, and spleen tissues were obtained for experiments. All experimental procedures were approved by the Ethics Committee of Suzhou Wuzhong People's Hospital.

### Evaluation of disease activity index

Mice were tested daily for body weight, gross rectal bleeding, and stool consistency to assess the extent of UC. Disease activity index (DAI) score was computed to quantify disease severity, as previously described (9,10).

### Hematoxylin and eosin staining

A colon segment (0.5 cm) was subjected to fixation with 10% formalin for 24 h. Subsequently, the colon segment was embedded with paraffin and sliced into 5-μm slices and then stained with hematoxylin-eosin (HE) for histological investigation.

### Spleen coefficient

The spleen coefficient was calculated as spleen coefficient = spleen weight (mg) / body weight (g).

### Immunohistochemistry (IHC)

Initially, the tissues were subjected to fixation with 4% paraformaldehyde. The tissue sections deparaffinized with xylene were hydrated with gradient ethanol and then incubated with F4/80 (ab6640, Abcam, China) antibody. Following the development utilizing diaminobenzidine, an optical microscope (Olympus BX51, Japan) was adopted for the observation of the sections.

### Enzyme-linked immunosorbent assay (ELISA)

The release of interleukin (IL)-6, IL-1β, and tumor necrosis factor-α (TNF-α) in the serum and the activities of myeloperoxidase (MPO), malondialdehyde (MDA), superoxide dismutase (SOD), and glutathione peroxidase (GSH-Px) in colon tissues were appraised by means of respective ELISA kits (Nanjing Jiancheng, China) following standard protocols.

### Reactive oxygen species (ROS) assay

ROS in colon tissues was identified using dihydroethidium (DHE) (Nanjing Jiancheng). A 0.5-cm colon segment was subjected to fixation with 10% formalin for 24 h. The colon segment was then embedded in paraffin, sliced into 5-μm slices, incubated with DHE dye (Servicebio, China) for 30 min at 37°C, and then stained with DAPI for 10 min. A fluorescence microscope was used to view the slides.

### Evaluation of the colonic permeability

With the purpose of evaluating colon permeability, serum levels of fluorescein isothiocyanate (FITC)-Dextran 4000 (FD-4) were determined. Mice were fasted for 20 h and then orally gavaged with FD-4 (60 mg/100 g). After 4 h, blood samples were collected and a multifunction microplate reader (BioTek Instruments, Inc., USA) was used to detect the level of FD-40 in serum in black 96-well plates at 490 nm of excitation wavelength and 520 nm of emission wavelength.

### Immunofluorescence (IF) assay

Colon tissues embedded with paraffin were cut at 4-μm thickness, followed by deparaffinization and rehydration. Subsequently, the antigen was extracted by microwave in 0.01 M sodium citrate buffer (pH=6.0) and then probed with primary Muc2 antibody (1:400, ab308191, Abcam) overnight at 4°C. On the second day, the sections were exposed to fluorescent-conjugated secondary antibody (1:400, ab150077, Abcam) at 37°C for 2 h, followed by 10 min of staining with DAPI. A fluorescence microscope was then used to photograph the sections.

### Western blot

The tissues were lysed on ice for 30 min and then centrifuged at 12,000 *g* at 4°C for 10 min to collect the supernatants. The BCA method (Bio‐Rad, USA) was employed for the quantification of protein concentration. Following the separation with 12% SDS‐PAGE, the proteins (30 µg/lane) were transferred to PVDF membranes. After blocking with 5% bovine serum albumin (BSA), the membranes were incubated with primary antibodies (1:1000; Abcam) targeting ANGPT2, ZO-1, Occludin, Claudin-1, p-mTOR, mTOR, p-p70S6K, p70S6K, LC3, LC3II, Beclin1, p62, NLRP3, GSDMD-N, IL-18, IL-1β, Caspase 1, or β-actin at 4°C overnight, following which they were exposed to horseradish peroxidase (HRP)-conjugated goat anti-rabbit IgG secondary antibodies (1:5000, Abcam) for 1 h. The visualization of protein bands was implemented with an Enhanced ECL Chemiluminescent Substrate Kit (Yeasen Biotechnology Co., Ltd., China), and the signal intensity was analyzed with ImageJ software (NIH, USA).

### Statistical analyses

GraphPad Prism analysis software (USA) was used. Data are reported as means±SD. For comparisons among multiple groups, one-way analysis of variance (ANOVA) followed by the Tukey's *post hoc* test were used.

## Results

### Silencing ANGPT2 improved the clinical symptoms and pathological changes in DSS-induced UC mice

**Figure 1:**
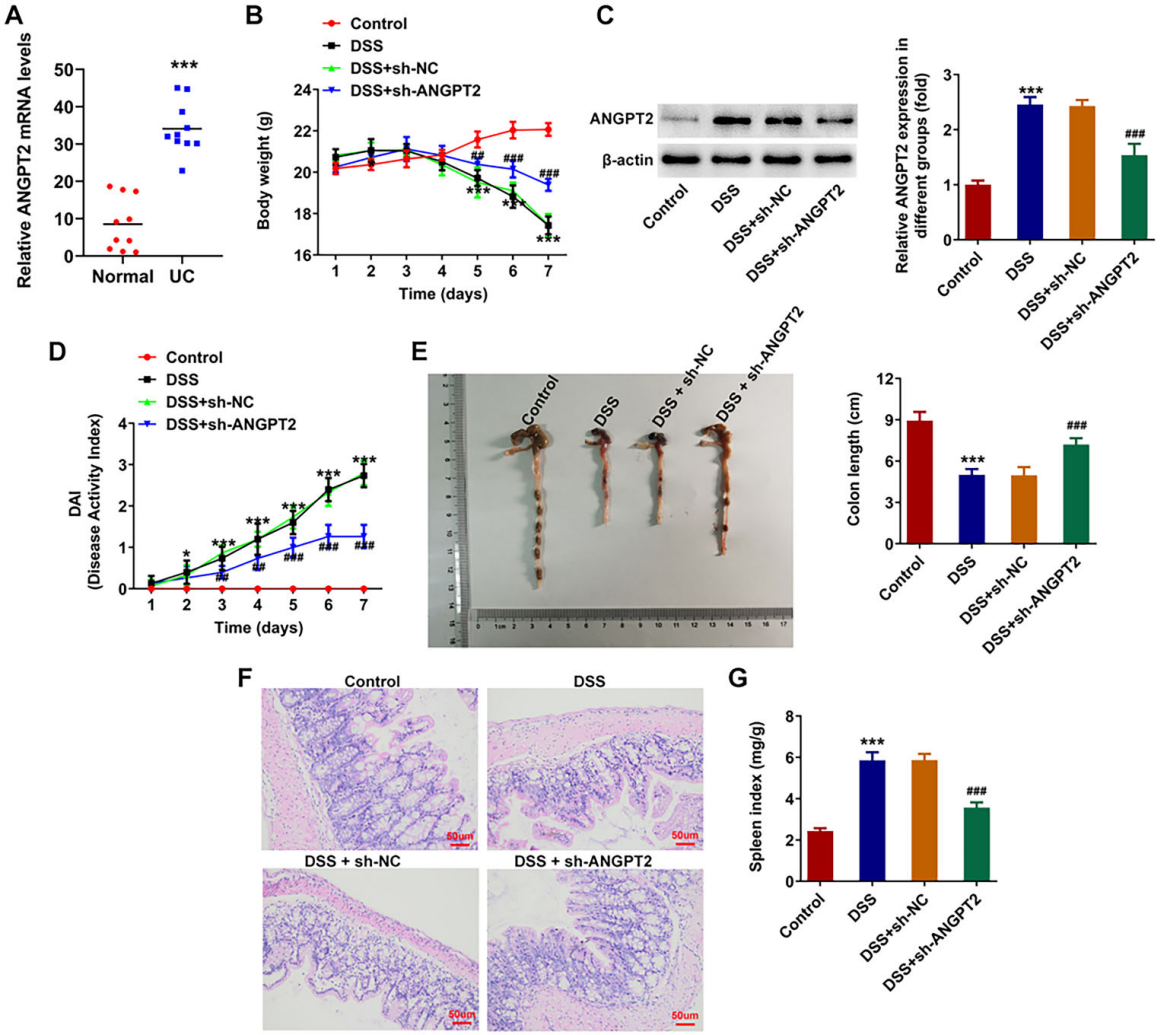
Silencing ANGPT2 improved clinical symptoms and pathological changes in dextran sodium sulfate (DSS)-induced ulcerative colitis (UC) mice. **A**, RT-qPCR was used to detect ANGPT2 level in serum of healthy participants and UC patients. ***P<0.001 *vs* Normal. **B**, Daily body weight of mice. **C**, Expression of ANGPT2 in the different groups. **D**, Disease activity index (DAI) score. **E**, Length of the colon of the groups. **F**, H&E staining was used to detect the pathological changes of the colon tissues (scale bar 50 μm). **G**, Spleen index of mice. *P<0.05, ***P<0.001 *vs* Control; ^##^P<0.01, ^###^P<0.001 *vs* DSS+sh-NC (negative control); ANOVA.

RT-qPCR was used to detect the ANGPT2 level in the serum of healthy participants and UC patients. ANGPT2 expression was significantly increased in serum of UC patients relative to the control group (Figure 1A). Relative to the control group, the body weight of mice treated by DSS was significantly decreased at the later stage of modeling, while that in the DSS + sh-ANGPT2 group was partially increased (Figure 1B). Results from western blot showed that the protein expression of ANGPT2 in DSS-treated mice was significantly increased in DSS group compared with the control group, which was then decreased by ANGPT2 knockdown (Figure 1C). Additionally, it was revealed that the DAI score of DSS-induced mice was significantly elevated, which was decreased with the depletion of ANGPT2 (Figure 1D). The colon length of DSS-induced mice was significantly shortened, while that in the DSS + sh-ANGPT2 group was greatly increased in comparison with the DSS + sh-NC group (Figure 1E). Histologically, the colon tissues of mice in DSS group were seriously damaged, while that of the DSS + sh-ANGPT2 group were partially alleviated (Figure 1F). The spleen index of mice treated by DSS was significantly elevated relative to the control group, and was reduced with the injection of sh-ANGPT2 (Figure 1G).

### Silencing ANGPT2 alleviated colonic inflammatory infiltration and oxidative stress in DSS-induced UC mice

**Figure 2:**
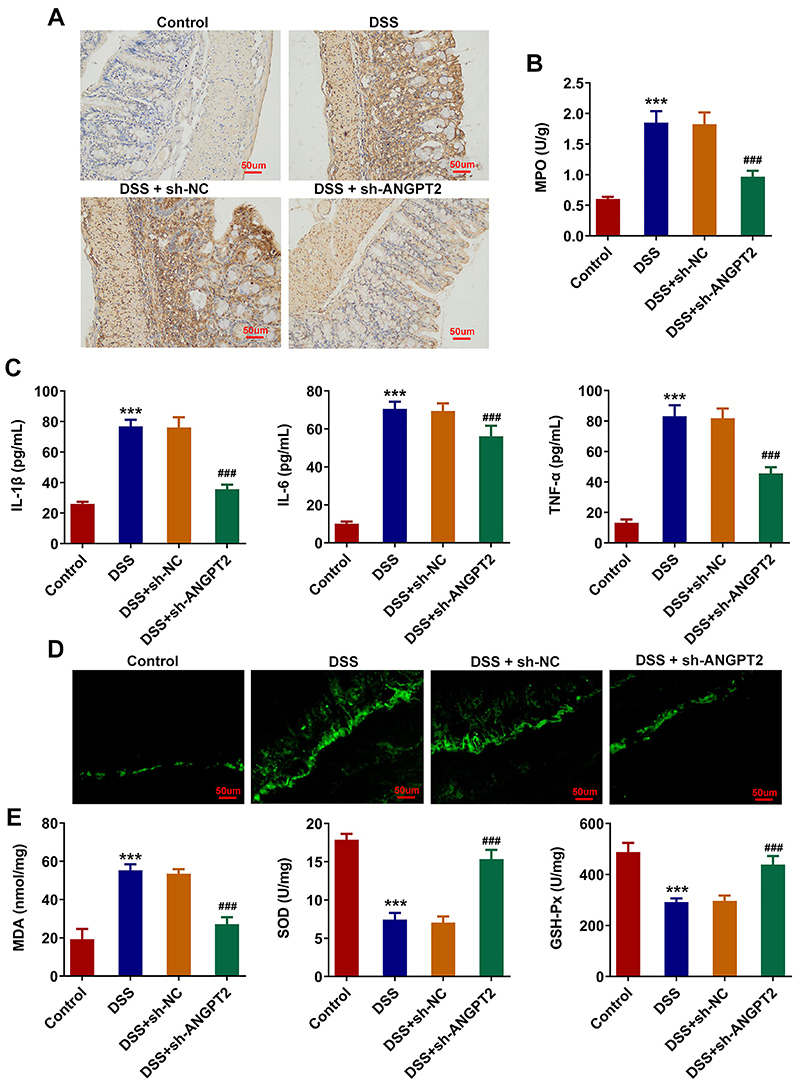
Silencing ANGPT2 alleviated colonic inflammatory infiltration and oxidative stress in dextran sodium sulfate (DSS)-induced ulcerative colitis (UC) mice. **A**, Immunohistochemical assay was used to detect the expression of F4/80 in macrophages in UC mice (scale bar 50 μm). **B**, MPO activity in colon tissues was detected by ELISA kit. **C**, Levels of inflammatory factors interleukin (IL)-1β, IL-6, and tumor necrosis factor (TNF)-α in serum. **D**, Reactive oxygen species (ROS) levels in colon tissues were detected by DHE staining (scale bar 50 μm). **E**, Oxidative stress markers malondialdehyde (MDA), superoxide dismutase (SOD), and glutathione peroxidase (GSH-Px) in colon tissues of the groups. ***P<0.001 *vs* control; ^###^P<0.001 *vs* DSS+sh-NC (negative control); ANOVA.

Results obtained from IHC assay demonstrated that F4/80 expression in macrophages in UC mice was increased, and was decreased by ANGPT2 knockdown (Figure 2A). MPO activity in DSS group was increased relative to that in control group, while ANGPT2 knockdown caused the opposite effects, evidenced by decreased MPO activity in DSS + sh-ANGPT2 group (Figure 2B). The levels of IL-6, IL-1β, and TNF-α were increased in the serum of UC mice, but decreased in DSS + sh-ANGPT2 group (Figure 2C). ROS content was elevated in the colon tissues of DSS-induced mice, and decreased in the DSS + sh-ANGPT2 group (Figure 2D). In addition, as shown in Figure 2E, DSS induction reduced the expressions of SOD and GSH-Px and increased MDA expression relative to those in the control group, which were then reversed by ANGPT2 interference.

### Silencing ANGPT2 maintained the colonic mucosal barrier in DSS-induced UC mice

**Figure 3:**
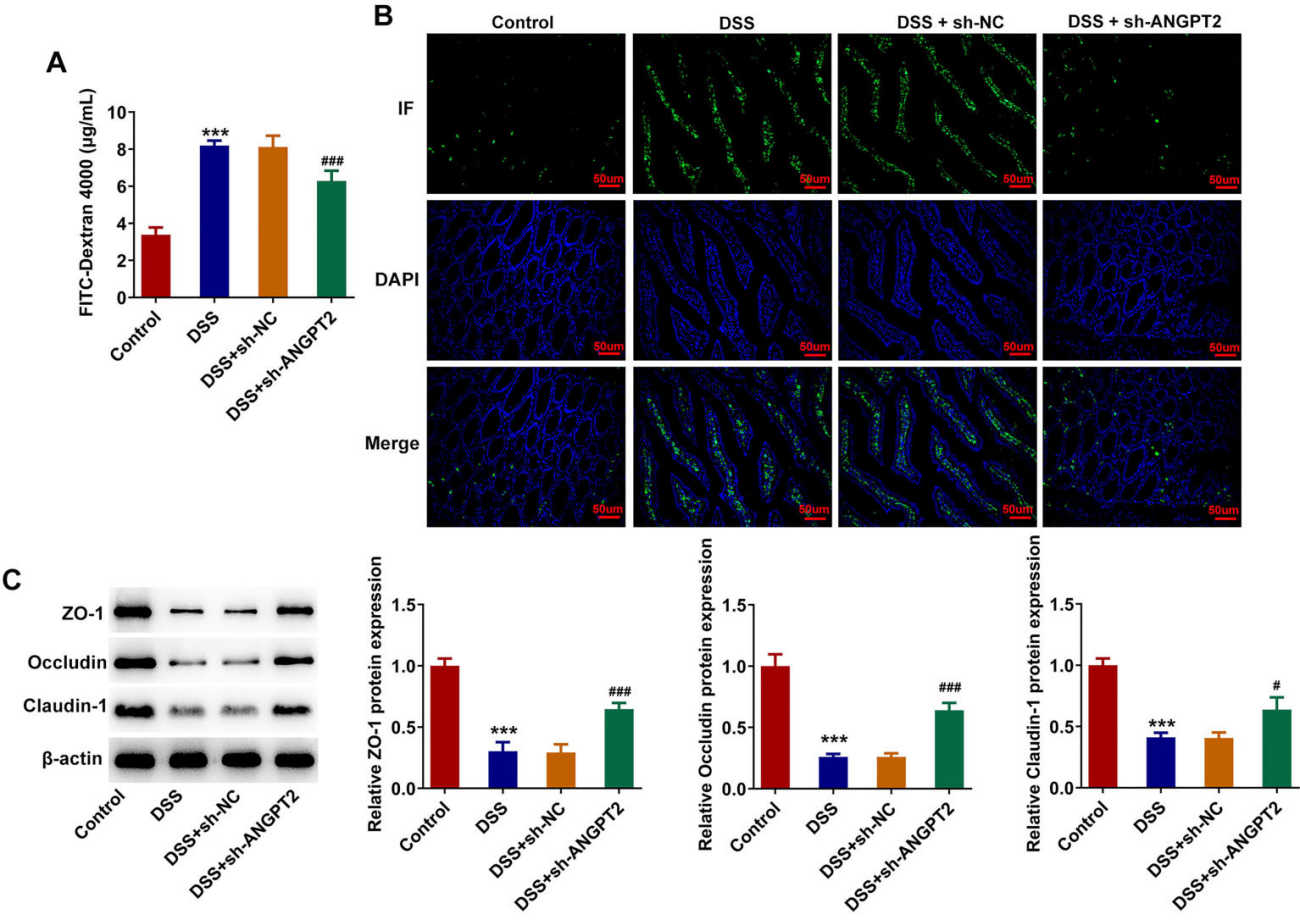
Silencing ANGPT2 maintained the colonic mucosal barrier in dextran sodium sulfate (DSS)-induced ulcerative colitis (UC) mice. **A**, The serum levels of fluorescein isothiocyanate (FITC)-Dextran 4000. **B**, Immunofluorescence assay was used to detect the expression of intestinal immunophenotypic marker Muc2 in colon tissues (scale bar 50 μm). **C**, Western blot detected the expressions of tight junction proteins ZO-1, Occludin, and Claudin-1 in colon tissues. ***P<0.001 *vs* Control; ^#^P<0.05, ^###^P<0.001 *vs* DSS+sh-NC (negative control); ANOVA.

As shown in Figure 3A, DSS induction significantly increased FD-4 expression compared to the control group, which was decreased by ANGPT2 deficiency. Subsequently, IF assay results showed that the expression of Muc2 was greatly reduced after DSS induction. Relative to the DSS + sh-NC group, Muc2 expression in DSS + sh-ANGPT2 group was significantly increased (Figure 3B). Results obtained from western blot showed that the contents of ZO-1, Occludin, and Claudin-1 were decreased in the DSS group relative to those in the control group, which were partially increased after depleting ANGPT2 (Figure 3C).

### Silencing ANGPT2 regulated the mTOR signaling pathway and thus affected autophagy-mediated NLRP3 inflammasome inactivation

**Figure 4:**
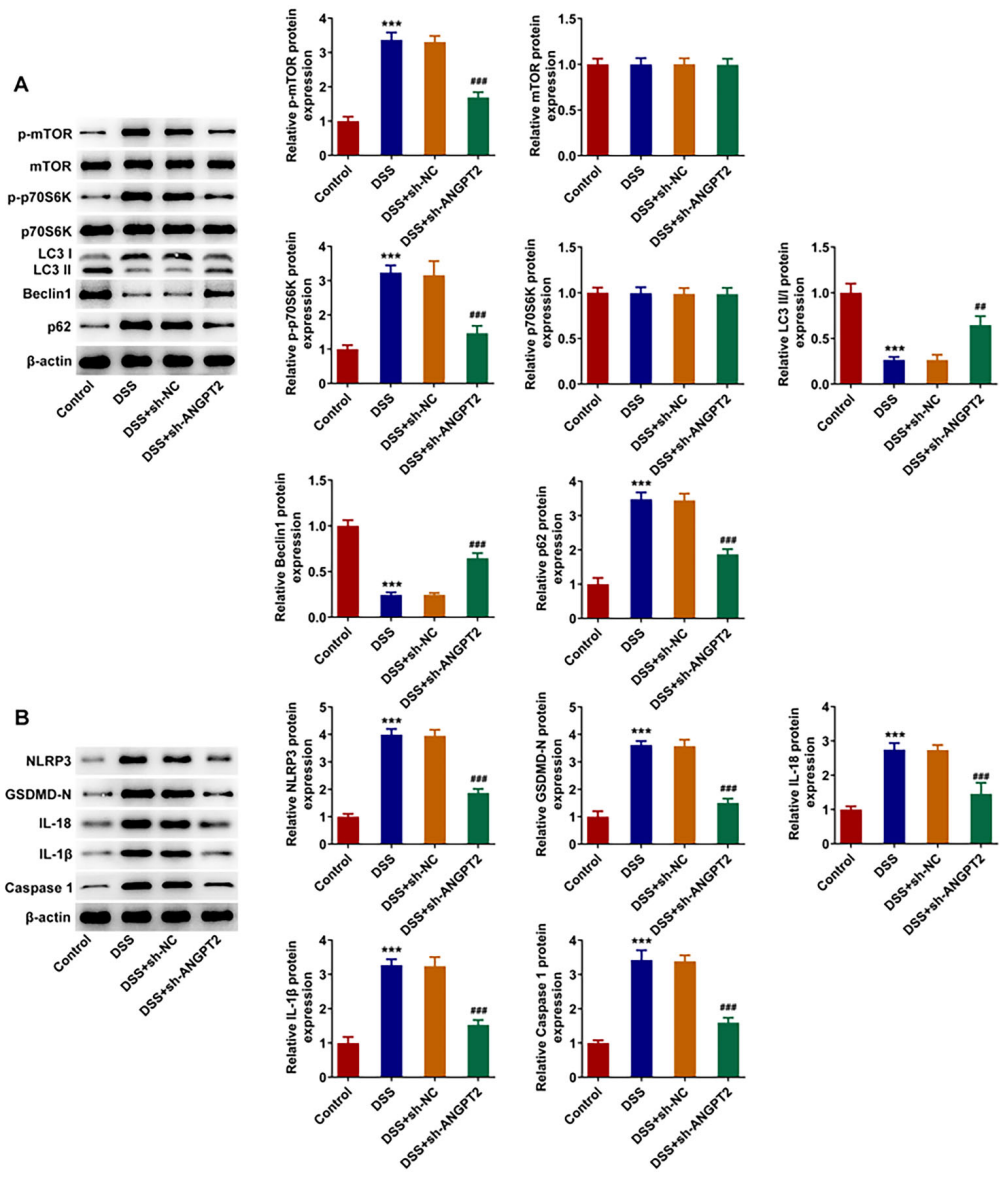
Silencing ANGPT2 regulated the mTOR signaling pathway and thus affected autophagy-mediated NLRP3 inflammasome inactivation in dextran sodium sulfate (DSS)-induced ulcerative colitis (UC) mice. Western blot analysis was used to detect the expressions of mTOR signaling pathway-related proteins (**A**) and the expressions of autophagy-related proteins (**B**). ***P<0.001 *vs* Control; ^##^P<0.01, ^###^P<0.001 *vs* DSS+sh-NC (negative control); ANOVA.

DSS induction significantly increased the expressions of p-mTOR, p-p70S6K, and P62 but decreased the expressions of LC3II/I and Beclin 1 compared with those in the control group, which were reversed by ANGPT2 silencing (Figure 4A). Western blot results demonstrated that relative to the control group, DSS induction increased the expressions of NLRP3, GSDMD-N, IL-18, IL-1β, and Caspase 1, which were subsequently reduced by ANGPT2 interference (Figure 4B).

## Discussion

Epidemiological studies have revealed that the global prevalence of UC is increasing (11). In UC, the immune response is impaired and the inflammatory signal is abnormal due to genetic susceptibility, epithelial barrier breakdown, and intestinal flora imbalance. Nevertheless, the exact etiology as well as the pathogenesis of UC is still poorly understood. Therefore, in this paper, the pathogenesis of UC was discussed, intending to seek effective treatment methods of UC.

ANGPT2 is an accurate early predictor of acute gastrointestinal injury and intestinal barrier dysfunction in patients with acute pancreatitis. It is superior to traditional biomarkers and can predict adverse outcomes and mortality caused by acute pancreatitis (12). Recombinant ANGPT1 reduces the expression of ANGPT2 induced by sepsis (13). ANGPT2 promotes inflammatory activation of monocytes in patients with systemic sclerosis (14). Accordingly, ANGPT2 has been suggested to play a certain regulatory role in inflammatory diseases. In our research, it was discovered that ANGPT2 expression in the serum of UC patients was significantly increased. With the aim of further determining the regulatory role of ANGPT2 in UC, ANGPT2 expression in UC mice was suppressed. The results demonstrated that ANGPT2 inhibition diminished the DAI score of UC mice, increased colon length, and improved the degree of colon lesions. Moreover, ANGPT2 silence alleviated colonic inflammatory infiltration and oxidative stress, and maintained the colonic mucosal barrier in DSS-induced UC mice.

Being a critical part of innate immunity, inflammasomes act as pivotal regulators in intestinal microbial infection, mucosal immune response, as well as metabolic process (15). Inflammasomes include NL-RP1, NLRP3, NLRC4, AIM2, NLRP6, and other subtypes, among which NLRP3 is one of the most comprehensively studied subtypes and is rapidly becoming an important regulator of intestinal homeostasis and inflammatory response (16,17). Despite the fact that targeting NLRP3 inflammasome is viewed as a promising therapy for UC, the regulatory mechanism of intestinal inflammation remains controversial.

It has been shown that the inhibition of ANGPT2 activates autophagy during hypertrophic scar formation through the PI3K/AKT/mTOR pathway (18). Autophagy plays a pivotal regulatory role in UC. Enhancing autophagy can alleviate intestinal mucosal barrier dysfunction in mice with colitis induced by glucan sodium sulfate (19). Lonicerin targeted EZH2 to attenuate UC through autophagy-mediated NLRP3 inflammasome inactivation (20). In addition, rapamycin ameliorated chronic intermittent hypoxia-induced kidney injury and sleep deprivation by regulating the mTOR/NLRP3 signaling pathway (21). Caffeine inhibited the activation of NLRP3 inflammasome through autophagy to reduce neuroinflammation-mediated by microglia cells in experimental autoimmune encephalomyelitis (22). Therefore, it is reasonable to speculate that ANGPT2 may regulate autophagy-mediated NLRP3 inflammasome through the mTOR signaling pathway in UC. In our experiments, we found that the depletion of ANGPT2 expression in UC mice suppressed the mTOR signaling pathway and triggered autophagy. In addition, we found that the inhibition of ANGPT2 in UC mice significantly reduced the expressions of inflammasome-related indicators NLRP3, GSDMD-N, IL-18, IL-1β, and Caspase 1. Our results suggested that ANGPT2 silence regulated the mTOR signaling pathway and thus affected autophagy-mediated NLRP3 inflammasome inactivation.

Our experiment also had some limitations. In terms of the exploration of the mechanism, mice were not administrated with any mTOR signaling pathway inhibitors to further prove our conclusions, which will be conducted in future experiments. In addition, the role of ANGPT2 in the cellular model of UC was not explored in this study, which will also be the focus of our future research.

### Conclusion

In this study, silencing ANGPT2 alleviated UC by regulating autophagy-mediated NLRP3 inflammasome inactivation via the mTOR signaling pathway. Our paper may provide theoretical feasibility for exploring the mechanism of the protein in the clinical treatment of UC.
